# Genes involved in carnitine synthesis and carnitine uptake are up-regulated in the liver of sows during lactation

**DOI:** 10.1186/1751-0147-55-24

**Published:** 2013-03-14

**Authors:** Susann Rosenbaum, Robert Ringseis, Erika Most, Sonja Hillen, Sabrina Becker, Georg Erhardt, Gerald Reiner, Klaus Eder

**Affiliations:** 1 Institute of Animal Nutrition and Nutritional Physiology, Justus-Liebig-University, Heinrich-Buff-Ring 26-32, Giessen, 35392, Germany; 2 Department of Veterinary Clinical Sciences, Swine Diseases, Justus-Liebig-University, Frankfurter Straße 112, Giessen, 35392, Germany; 3 Institute for Animal Breeding and Genetics, Justus-Liebig-University, Ludwigstraße 21b, Giessen, 35390, Germany

**Keywords:** Lactation, Sow, Liver, PPARα pathway, Carnitine

## Abstract

**Background:**

Convincing evidence exist that carnitine synthesis and uptake of carnitine into cells is regulated by peroxisome proliferator-activated receptor α (PPARA), a transcription factor which is physiologically activated during fasting or energy deprivation. Sows are typically in a negative energy balance during peak lactation. We investigated the hypothesis that genes involved in carnitine synthesis and uptake in the liver of sows are up-regulated during peak lactation.

**Findings:**

Transcript levels of several PPARα target genes involved in fatty acid uptake (FABP4, SLC25A20), fatty acid oxidation (ACOX1, CYP4A24) and ketogenesis (HMGCS2, FGF21) were elevated in the liver of lactating compared to non-lactating sows (P < 0.05). In addition, transcript levels of genes involved in carnitine synthesis (ALDH9A1, TMLHE, BBOX1) and carnitine uptake (SLC22A5) in the liver were greater in lactating than in non-lactating sows (P < 0.05). Carnitine concentrations in liver and plasma were about 20% and 50%, respectively, lower in lactating than in non-lactating sows (P < 0.05), which is likely due to an increased loss of carnitine via the milk.

**Conclusions:**

The results of the present study show that PPARα is activated in the liver of sows during lactation which leads to an up-regulation of genes involved in carnitine synthesis and carnitine uptake. The PPARα mediated up-regulation of genes involved in carnitine synthesis and uptake in the liver of lactating sows may be regarded as an adaptive mechanism to maintain hepatic carnitine levels at a level sufficient to transport excessive amounts of fatty acids into the mitochondrion.

## Findings

Lactation is associated with a dramatic increase in the energy and nutrient requirement of the organism for milk production which is usually met by an elevation of food intake, a mobilisation of body’s energy stores, and several metabolic adaptations [1,2]. In order to improve the knowledge about metabolic adaptations during lactation in sows we have recently analyzed the changes in the hepatic transcriptome of sows during lactation using a porcine whole-genome microarray [3]. Interestingly, enrichment analysis using the Kyoto Encyclopedia of Genes and Genomes (KEGG) database revealed that peroxisome proliferator-activated receptor α (PPARα) signalling is one of the regulatory pathways which is activated in the liver of sows during lactation [3]. PPARα is a ligand-activated transcription factor which induces of a large set of genes encoding proteins involved in fatty acid catabolism, such as fatty acid activation, fatty acid β-oxidation, fatty acid hydroxylation, ketogenesis and gluconeogenesis [4,5]. This transcription factor is known to be activated by non-esterified fatty acids (NEFA) [4,5], which are released from adipose tissue during early lactation due to the strong increase in the sow’s energy requirement for milk production which cannot be completely covered by an increase of feed intake [3]. Genes involved in fatty acid catabolism are up-regulated by PPARα because the regulatory regions of these genes contain specific DNA sequences, called peroxisome proliferator response elements (PPRE), which are bound by the activated PPARα complex and mediate transcription of these genes [4,5]. Studies in mice, rats and pigs indicated that PPARα is not only a critical regulator of fatty acid catabolic genes but also a key regulator of genes involved in carnitine synthesis and uptake [6]. In pigs, carnitine biosynthesis occurs exclusively in liver and kidney, with liver being quantitatively most important [7]. Convincing evidence for the regulation of genes involved in carnitine synthesis and uptake by PPARα has been provided from genetic studies in which functional PPRE have been identified in the regulatory regions of the mouse genes encoding the carnitine transporter novel organic cation transporter 2 (OCTN2/SLC22A5) and two enzymes of the carnitine biosynthetic pathway, γ-butyrobetaine dioxygenase (BBOX1) and 4-trimethylaminobutyraldehyde dehydrogenase (ALDH9A1) [8-10]. Like in lactating sows, activation of hepatic PPARα and up-regulation of fatty acid catabolic genes in the liver has been reported in early lactating cows [11-13]. In addition, induction of genes involved in carnitine synthesis and uptake (SLC22A5, BBOX1, ALDH9A1) in the liver and a marked increase in the hepatic carnitine concentration has been observed in cows during lactation [14]. The increase in the hepatic concentration of carnitine in the early lactating cow has been interpreted as a means to supply the liver with sufficient carnitine required for transport of excessive amounts of fatty acids into the mitochondrion. Regarding that PPARα is activated and genes involved in fatty acid catabolism are up-regulated also in the liver of sows during lactation, we tested the hypothesis that genes involved in carnitine synthesis and uptake are up-regulated in the liver of sows during peak lactation.

The animal study was conducted in accordance with established guidelines for the care and handling of laboratory animals and was approved by the local Animal Welfare Authorities (Regierungspräsidium Giessen; permission no: GI 19/3-No. 29/2010). The experiment included twenty second parity sows (Large White & German Landrace), which were artificially inseminated with semen from boars of the own breed, and kept in single crates until day 21 of pregnancy. From day 21 to 110 of pregnancy the sows were kept in groups in pens that had fully slatted floors, nipple drinkers and feeding stations, and from day 110 of pregnancy until farrowing the sows were housed in single farrowing pens. During pregnancy, all sows received a commercial diet for gestating sows ad libitum. 24 h after farrowing, the sows were randomly assigned into two groups of 10 animals each. In the first group of sows designated as “non-lactating group” (average body weight: 259 ± 17 kg), all piglets were removed from the sow 24 h after farrowing. In the second group of sows designated as “lactating group” (average body weight: 256 ± 17 kg), litters were standardised to 12 piglets per sow. To ensure an adequate temperature of 35°C for the piglets an infrared heater was placed directly above the site of the newborn piglets. The temperature and the relative humidity in the dry sow accommodation and the farrowing unit were kept at 19 ± 1°C and 60–80%, respectively, by means of an air conditioning system. In addition, a light:dark cycle of 12-h light and 12-h dark was applied. During lactation until the end of the experiment the sows were given a diet for lactating sows. Until day 6 of lactation, the amount of feed given to the lactating sows was successively increased (1.6 kg/d on day 1; 2.6 kg/d on day 2; 4.1 kg/d on day 3; 5 kg/d on day 4; 5.5 kg/d on day 5 and 6.0 kg/d on day 6), and from day 7 of lactation and thereafter the sows received individual amounts of feed depending on their body weights. In the non-lactating group, each sow was given an amount of food sufficient to cover the individual energy and nutrient requirement for maintenance.

The diet for gestating sows consisted of (in g/kg diet): wheat (160), barley (705), soy bean meal with 43% crude protein (80), soy oil (5) and a mineral supplement (50) (Sano Fasersan Trag®, Sano-Moderne Tierernährung GmbH, Loiching, Germany) and had a metabolizable energy of 12.2 MJ per kg diet. The diet for lactating sows consisted of (in g/kg diet): wheat (440), barley (350), soy bean meal with 43% crude protein (160), soy oil (15) and a mineral supplement (35) (Sauengold Lac®, Sano-Moderne Tierernährung GmbH, Loiching, Germany) and had a metabolizable energy of 13.1 MJ per kg diet. Both types of diets (gestation and lactation) were not supplemented with carnitine. The native carnitine concentration of the diets was very low (< 5 mg/kg diet) as determined by tandem mass spectrometry (see below).

On day 20 after farrowing, blood was collected from *V. jugularis* 3 h after feed intake in heparinised polyethylene tubes (Sarstedt, Nürnberg, Germany) and plasma was obtained by centrifugation and stored at −20°C. Liver samples were taken by biopsy which took place in a cleaned and disinfected room in the animal keeping facility. Anesthetization of the sows was carried out as described recently in detail [3]. The liver biopsy samples were taken percutaneously in the left lateral cumbency with a 16 G/1.65 mm biopsy needle (length: 160 mm) on a HistoCore® system (BIP Biomed. Instrumente & Produkte GmbH, Germany) as described elsewhere [15]. Liver samples were immediately snap-frozen and stored at −80°C until analysis. Total RNA from frozen liver samples was isolated using TrizolTM reagent (Invitrogen, Karlsruhe, Germany) according to the manufacturer’s protocol and purified using the RNeasy Minikit (Qiagen, Hilden, Germany). cDNA synthesis and quantitative real-time PCR (qPCR) analysis were performed as described recently in detail [16]. Characteristics of primers and primer performance data used for qPCR analysis are shown in Table 1. Concentrations of free carnitine and acetylcarnitine in plasma, liver and diets were determined by tandem mass spectrometry according to Hirche et al. [17]. Statistical analysis was performed by one way analysis of variance. Fisher’s multiple range test was used to generate significant F-values of differences with P < 0.05.

**Table 1:** Characteristics of primers and primer performance data used for qPCR

**Gene symbol**	**Forward primer (from 5**^**′ **^**to 3**^**′**^**)**	**Product size (bp)**	**NCBI GenBank**	**Slope**	**R2**^**#**^	Efficiency*	**M**
	**Reverse primer (from 5**^**′ **^**to 3**^**′**^**)**						
*Reference genes*							
RSP9	GTCGCAAGACTTATGTGACC	325	XM_003356050	−0.28	0.999	1.91	0.053
	AGCTTAAAGACCTGGGTCT						
ATP5G1	CAGTCACCTTGAGCCGGGCGA	94	NM_001025218	−0.30	0.998	1.99	0.054
	TAGCGCCCCGGTGGTTTGC						
GSR	AGCGCGATGCCTACGTGAGC	175	AY368271	−0.29	0.997	1.94	0.055
	GGTACGCCGCCTGTGGCAAT						
ACTB	GACATCCGCAAGGACCTCTA	205	XM_003124280	−0.32	0.992	2.10	0.064
	ACATCTGCTGGAAGGTGGAC						
SHAS2	GAAAAGGCTAACCTACCCTG	218	NM_214053	−0.21	0.996	1.65	0.076
	TGTTGGACAAGACCAGTTGG						
*Target genes*							
ACOX1	CTCGCAGACCCAGATGAAAT	218	AF185048	−0.28	0.995	1.90	
	TCCAAGCCTCGAAGATGAGT						
ALDH9A1	GCTGCTGGCCGAAATCTATA	215	XM_001924860	−0.29	0.999	1.94	
	CACACTTCCAGTGAAGGAGA						
BBOX1	GTGCCGAAAGCTCAAGGAAAAA	342	XM_003122909	−0.31	0.985	2.06	
	CTCTGCCGGCCGTGAAGTAAC						
CYP4A24	GGTTTGCTCCTGTTGAATGG	121	NM_214424	−0.29	0.999	1.96	
	GCATCACTTGGACAGACTTG						
FABP4	CACCAGGAAGGTGGCTGGCA	197	NM_001002817	−0.30	0.999	1.98	
	CCTGTACCAGGGCGCCTCCA						
FGF21	GAAGCCAGGGGTCATTCAAA	149	NM_001163410	−0.31	0.999	2.03	
	GGTAAACGTTGTAGCCATCC						
HMGCS2	GGACCAAACAGACCTGGAGA	198	NM_214380	−0.32	0.994	2.07	
	ATGGTCTCAGTGCCCACTTC						
SLC22A5	TGACCATATCAGTGGGCTA	384	XM_003123912	−0.30	0.995	2.00	
	AGTAGGGAGACAGGATGCT						
SLC25A20	GCAAAGCCCATTAGCCCTCT	312	XM_003483178	−0.28	0.998	1.89	
	GAGCACATCCTCTGGGTGTT						
TMLHE	GCACCATACAGCCTCCAAGT	221	XM_003135511	−0.35	0.995	1.79	
	TGGTCTCATCCAGACGAACA						

^#^Coefficient of determination of the standard curve. *The efficiency is determined by [10^-slope^].

Like in dairy cows, the transition from pregnancy to lactation in sows is associated with severe metabolic adaptations. The production of milk causes a strong increase of the sow’s energy requirement which however cannot be completely covered by an increase of feed intake. Indeed, the sows of the lactating group exhibited a greater loss of body weight from day 1 to day 20 of lactation than sows of the non-lactating group, despite showing a markedly greater feed intake [3]. In line with this, we found that the sows of the lactating group are in a strong negative energy balance during this period [3]. To compensate the negative energy balance NEFA are typically mobilized from adipose tissue which was demonstrated in the present study by elevated plasma levels of NEFA in the lactating compared to the non-lactating sows [3]. NEFA are then transported via the circulation to the tissues, mainly the liver, from which they are taken up and where they bind to and activate PPARα [18]. In agreement with this, we observed in the present study that the negative energy balance in sows of the lactating group was associated with strongly elevated (2 to 10-fold) transcript levels of several PPARα target genes involved in fatty acid uptake (FABP4, SLC25A20), fatty acid oxidation (ACOX1, CYP4A24) and ketogenesis (HMGCS2, FGF21) (P < 0.05, Table 2). Given that the above mentioned genes contain functional PPRE motifs in their regulatory regions [5], the up-regulation of these genes during lactation indicates, at least indirectly, that the PPARα pathway is indeed activated in the liver of lactating sows. Due to the limited amount of liver tissue obtained from the biopsy sampling procedure additional assays, such as EMSA, providing direct evidence for activation of PPARα could not be conducted. Due to the same reason protein levels of the PPARα target genes were not determined. However, several studies clearly showed that increased mRNA levels of PPARα target genes positively correlate with elevated levels of the encoded proteins [19], and are therefore suitable indicators of PPARα activation.

**Table 2:** Relative transcript levels of classical PPARα target genes involved in fatty acid uptake, fatty acid oxidation and ketogenesis in the liver of lactating and non-lactating sows on day 20 of lactation

	**Non-lactating (n = 10)**	**Lactating (n = 10)**
*Fatty acid uptake*		
FABP4	1 ± 0.36	1.88 ± 0.87*
SLC25A20	1 ± 0.60	4.63 ± 1.02*
*Fatty acid oxidation*		
ACOX1	1 ± 0.59	2.43 ± 1.47*
CYP4A24	1 ± 0.41	2.14 ± 1.47*
*Ketogenesis*		
FGF21	1 ± 0.70	4.86 ± 2.25*
HMGCS2	1 ± 1.14	9.63 ± 6.67*

Values represent mean ± SD for n = 10 sows per group. *Significantly different from non-lactating group (P < 0.05).

In accordance with the hypothesis of this study, we found for the first time that lactation causes an up-regulation of genes involved in carnitine synthesis (ALDH9A1, TMLHE, BBOX1) and carnitine uptake (SLC22A5) in the liver of sows (P < 0.05, Table 3). Considering that at least the mouse genes encoding ALDH9A1, BBOX1 and SLC22A5 were shown to be direct PPARα target genes [8-10], it is very likely that the up-regulation of these genes in the liver of lactating sows was caused by the activation of PPARα. This assumption is strongly supported by the fact that the functional PPRE identified in the mouse SLC22A5 gene is completely identical (100%) and the functional PPREs of mouse ALDH9A1 and BBOX1 show a high similarity between mouse and pig [6]. In addition, treatment with the synthetic PPARα activator clofibrate [20] and food deprivation [21] was reported to cause a marked up-regulation of SLC22A5 and BBOX1 and an increase in the activity of butyrobetaine dioxygenase (encoded by BBOX1), the rate-limiting enzyme of carnitine synthesis, in the liver of pigs. Moreover, treatment with clofibrate [20] and food deprivation [21] causes a significant increase in the hepatic concentration of carnitine in pigs. In the present study we found that the carnitine concentration in the liver was approximately 20% lower in lactating than in non-lactating sows (Free carnitine: 43.88 ± 9.13 vs. 56.1 ± 11.5 nmol/g wet weight, n = 10/group, P < 0.05), despite the strong increase in the expression of genes involved in carnitine synthesis and uptake. In addition, the concentration of carnitine in plasma was even about 50% lower in lactating than in non-lactating sows (Free carnitine: 6.71 ± 0.63 vs. 12.90 ± 0.98 μmol/L, Acetylcarnitine: 0.73 ± 0.16 vs. 1.53 ± 0.20 μmol/L, n = 10/group, both P < 0.001), which is likely due to loss of carnitine from the body pool via the milk. Notably, milk of sows is rich in carnitine. Carnitine concentrations in sows’ milk were reported to be in the range between 120 and 185 μmol/L, depending on the stage of lactation [22,23]. Thus, production of 8–10 liter of milk per day is associated with a loss of 1 to 2 mmoles of carnitine, an amount which is in great excess of the whole plasma carnitine pool. Thus, mobilisation of carnitine from tissue stores and an increase in carnitine synthesis rate might be important for supplying carnitine for milk. These observations, therefore, suggest that the PPARα mediated up-regulation of genes involved in carnitine synthesis and uptake in the liver of lactating sows is an adaptive mechanism to maintain hepatic carnitine levels at a level sufficient to transport excessive amounts of fatty acids into the mitochondrion. However, the lower hepatic carnitine level in the liver of lactating than in non-lactating sows indicates that the increase in carnitine synthesis and uptake in the liver of lactating sows cannot completely compensate the loss of carnitine via the milk.

**Table 3:** Relative transcript levels of genes involved in carnitine synthesis and carnitine uptake in the liver of lactating and non-lactating sows on day 20 of lactation

	**Non-lactating (n = 10)**	**Lactating (n = 10)**
*Carnitine uptake*		
SLC22A5	1 ± 0.24	1.54 ± 0.51*
*Carnitine synthesis*		
ALDH9A1	1 ± 0.55	2.72 ± 1.04*
TMLHE	1 ± 0.41	1.54 ± 0.34*
BBOX1	1 ± 0.43	2.97 ± 0.96*

Values represent mean ± SD for n = 10 sows per group. *Significantly different from non-lactating group (P < 0.05).

Previous studies have shown that supplementation of carnitine to sows during pregnancy offers beneficial effects on fetal growth and muscle development, number of piglets born and piglet and litter weights at birth [24-27]. It has been assumed that these effects of carnitine were mediated by influencing the secretion of IGF-1 and insulin and by an increased intrauterine nutrition of fetuses [24,25,28]. In contrast, supplementation of carnitine during lactation had less effects on sow and litter performance [22,24]. Thus, our observation of an increased expression of enzymes of carnitine synthesis in the liver during lactation, indicative of an increased carnitine biosynthesis, could provide an explanation for the opposite effects of carnitine supplementation in pregnancy and lactation.

In conclusion, the results of the present study show that the PPARα pathway is activated in the liver of sows during lactation which leads to an up-regulation of genes involved in carnitine synthesis and carnitine uptake. The PPARα mediated up-regulation of genes involved in carnitine synthesis and uptake in the liver of lactating sows may be regarded as an adaptive means to maintain hepatic carnitine levels at a level sufficient to transport excessive amounts of fatty acids into the mitochondrion.

## Competing interests

The authors declare that they have no competing interests.

## Authors’ contributions

SR conducted the animal experiment, performed the PCR analyses and the statistical analyses and wrote the manuscript. RR supervised PCR analyses and helped to draft the manuscript. SH and GR established the liver biopsy sampling procedure. SL conducted liver biopsy sampling. GE was responsible for animal keeping. KE conceived of the study, participated in its design and coordination and helped to draft the manuscript. All authors read and approved the final manuscript.
